# Replacement‐Based Ageing Interventions for Systemic Rejuvenation: Shaping Longevity Science and Clinical Directions

**DOI:** 10.1111/acel.70516

**Published:** 2026-04-29

**Authors:** Bjorn Fraser Olaisen, Vadim N. Gladyshev, Bohan Zhang, Anthony Atala, Kyle M. Loh, Vera Gorbunova, Thomas A. Rando, Tony Wyss‐Coray, Eric Verdin, Alexander Zhavoronkov, Morten Scheibye‐Knudsen, Sierra Lore, Daniela Bakula

**Affiliations:** ^1^ University of Cambridge Cambridge UK; ^2^ Brigham and Women's Hospital Harvard Medical School Boston Massachusetts USA; ^3^ Broad Institute of MIT and Harvard Cambridge Massachusetts USA; ^4^ Wake Forest Institute for Regenerative Medicine Winston‐Salem North Carolina USA; ^5^ Stanford University Stanford California USA; ^6^ University of Rochester Rochester New York USA; ^7^ University of California Los Angeles California USA; ^8^ Buck Institute for Research on Aging Novato California USA; ^9^ Insilico Medicine US Inc Boston Massachusetts USA; ^10^ University of Copenhagen Copenhagen Denmark

## Abstract

Biological and synthetic replacement‐based ageing interventions hold substantial potential to reverse many forms of age‐related damage simultaneously and extend healthy lifespan beyond what can be achieved with conventional therapeutics. In this Perspective, we discuss recent insights, unmet needs, and emerging trajectories that are catalysing research and clinical development of replacement‐based treatments and synergistic strategies for multi‐targeted damage removal and export at the molecular, organellar, and cellular levels. The first workshop dedicated to replacement as an ageing intervention at the Aging Research & Drug Discovery 2025 conference helped prioritise key challenges, opportunities, and future directions to address the need for preventive replacement and bioengineering technologies capable of inducing systemic and sustained rejuvenation across cells, tissues, and regulatory networks. We propose a roadmap to guide research and innovation integrating replacement and next‐generation damage‐removal therapeutics to modulate the ageing process in the whole body, restore biological function, and extend healthy lifespan.

## Graphical abstract

We propose a roadmap to guide the development of rejuvenation therapeutics integrating replacement‐based ageing interventions, regeneration, and synthetic technologies that remove or export hundreds of types of molecular, organellar, and cellular damage. Recommended bioengineering strategies would supersede endogenous repair and enable systemic, whole‐body rejuvenation aimed at substantially extending healthy lifespan.

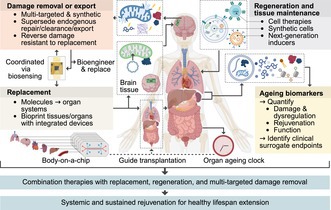

Recent discoveries and therapeutic developments in longevity science have made it increasingly clear that a vast amount of age‐related damage and systemic changes at the level of molecules, organelles, cells, tissues, organs, and organisms must be reversed to yield durable and multi‐tissue rejuvenation as well as further extensions in healthy lifespan. Advanced biological and synthetic replacement, maintenance, and multi‐targeted damage‐removal strategies for cells, tissues, and organ systems represent some of the most promising longevity interventions, with the potential to reverse an unprecedented fraction of age‐related changes, and to prevent, slow, or even reverse age‐related diseases and dysfunction (Lore et al. 2025).

We define replacement‐based ageing interventions as strategies that replace cells, tissues, organs, physiological systems, or cellular components (e.g., mitochondria or genes) with biological or synthetic alternatives. Biological replacements include transplantation of stem cells, organs, and bioprinted tissues (e.g., progressive brain replacement using cells and biomaterials), bioengineered cell therapies (e.g., CAR‐T and synthetic cells), and therapeutic plasma exchange. Synthetic replacements include prostheses, external medical devices (e.g., dialysis machines), and brain–machine interface systems (e.g., cortical implants) (Lore et al. 2025).

Replacement‐based approaches are predicted to act synergistically with emerging regeneration and synthetic damage‐removal technologies capable of targeting and exporting hundreds of molecular and organellar damage types from cells without relying solely on inherently limited and declining endogenous repair, clearance, and export processes.

## 1 The First Replacement in Aging Workshop

**FIGURE 1:**
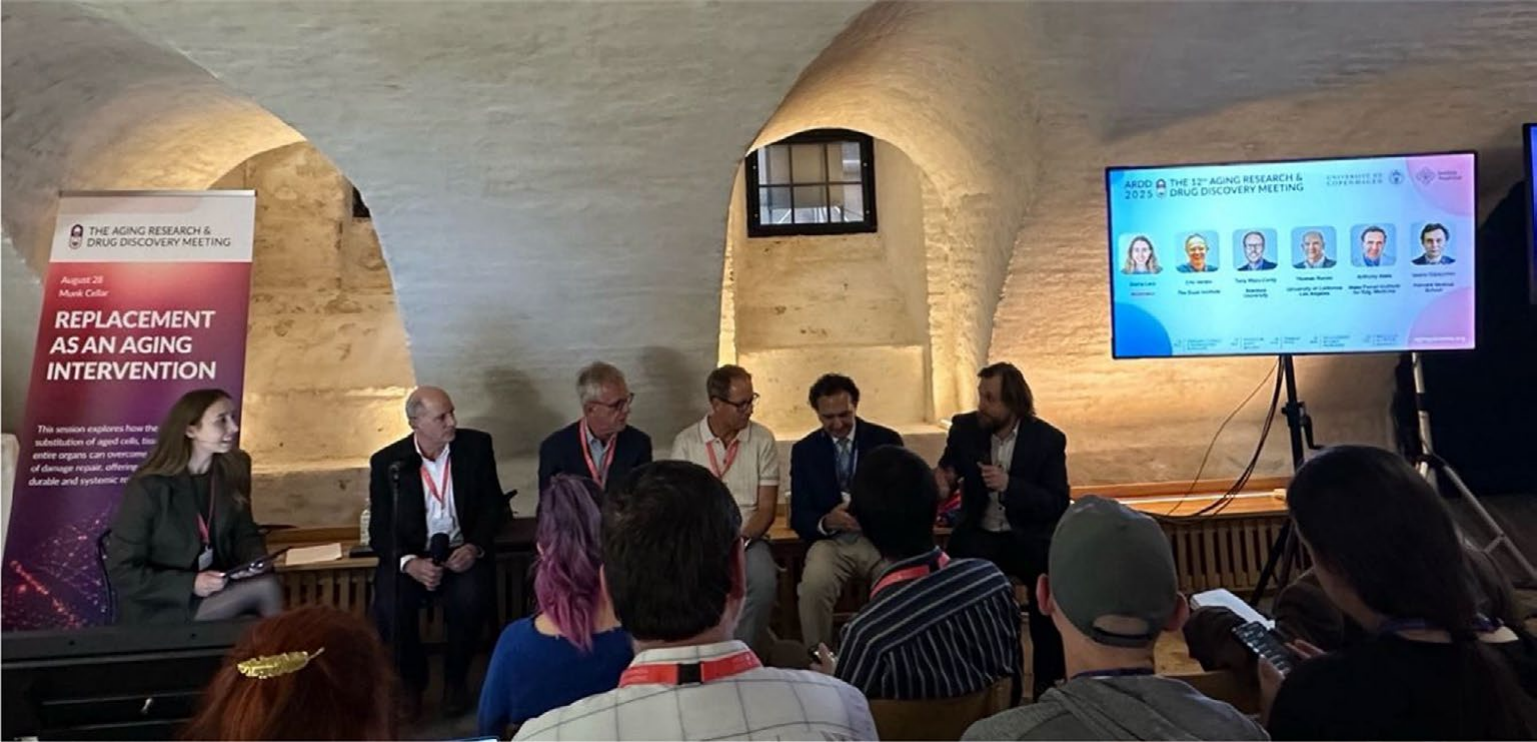
Panel discussion on predicted and recommended advances for replacement‐based ageing interventions. From the left: Sierra Lore (Moderator, University of Copenhagen and Buck Institute for Research on Aging), Thomas A. Rando (University of California, Los Angeles), Eric Verdin (Buck Institute for Research on Aging), Tony Wyss‐Coray (Stanford University), Anthony Atala (Wake Forest Institute for Regenerative Medicine), and Vadim N. Gladyshev (Harvard Medical School). Photo: Bjorn Fraser Olaisen (University of Cambridge).

**FIGURE 2:**
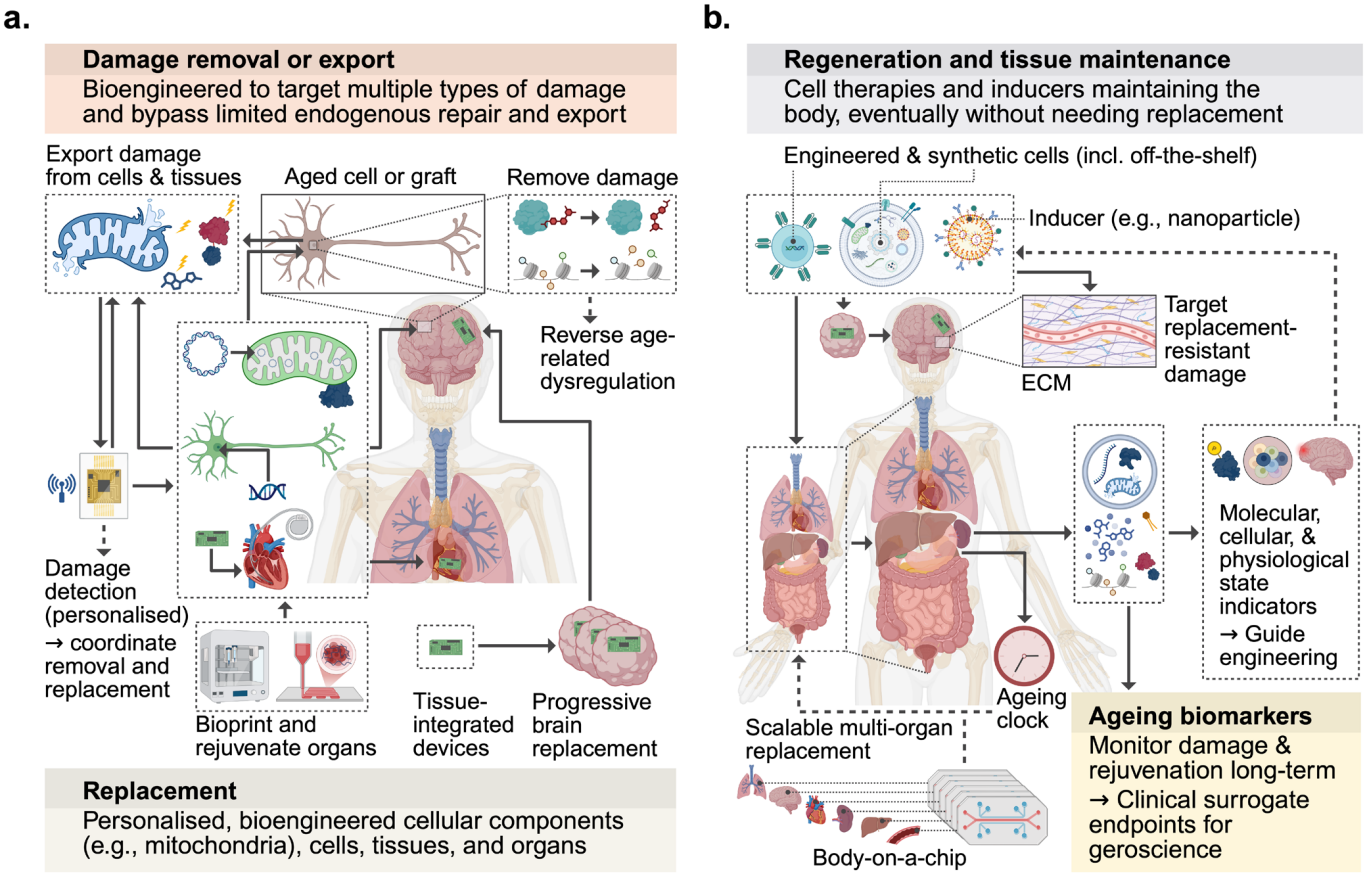
Prioritised rejuvenation strategies integrating systemic replacement, regeneration, and damage removal or export. There is a need for rejuvenation therapies that combine replacement strategies with the removal or export of damaged molecules (e.g., aggregated proteins) and organelles (e.g., mitochondria) from cells and tissues (a), in addition to personalised cell therapies and inducers that maintain and regenerate the body (b), and technologies that reverse age‐related changes resistant to replacement. The development of novel biomarkers capable of capturing long‐term rejuvenation across molecules, cells, tissues, organs, and whole systems could provide surrogate endpoints for clinical trials targeting ageing. Created in https://www.biorender.com/. https://BioRender.com/8rrerid.

In line with the recent perspective on replacement in ageing (Lore et al. 2025), and the Aging Research and Drug Discovery meeting's focus on scientific discoveries and clinical translation in longevity science (Dekan et al. 2026), the Replacement in Aging workshop (Figure 1) aimed to prioritise and catalyse research trajectories and clinical avenues focused on developing replacement‐based ageing interventions for systemic and sustained rejuvenation. In this report, we reflect on challenges (Table 1a), opportunities (Table 1b), and the vision of the growing subfield of replacement in ageing (Table 2), including how replacement can be combined with synergistic regeneration and damage‐removal strategies (Figure 2a,b).

**TABLE 1:** Recommended research directions for replacement in ageing.

a. Key challenges for replacement‐based ageing interventions
Systemic and durable rejuvenation Extensive multi‐level damage and dysregulation (molecular to systemic) Limited long‐term vision for preventive/systemic replacement Lack of cross‐organ, whole‐body rejuvenation frameworks Brain replacement with preserved self‐identity
Ageing biomarkers and surrogate endpoints Limited biomarkers for cross‐intervention rejuvenation Long timelines for clinical validation Cell‐ and organ‐specific intervention responses
Transplantation therapies Rapid graft ageing (i.e., “age assimilation”) Transplantation induces damage and transient, limited rejuvenation Number of organs required for systemic rejuvenation Dependence on lifelong immunosuppression for allogeneic replacement Age‐ and immune‐related incompatibilities (e.g., host infiltration) Risk of HSC transplantation mortality Excessive plasma transfusions limit age reduction
Bioengineering and novel replacement strategies Slow, complex engineering of solid organs Limited vascularisation of engineered human tissues Co‐culturing multiple cell types Reliance on autologous primary cells Inefficient multi‐gene/cell delivery

b. Emerging research opportunities for replacement‐based ageing interventions
Systemic and durable rejuvenation Multi‐targeted damage removal and export technologies (e.g., mitochondria) Target changes not fully reversed by replacement (e.g., ECM remodelling) Preventive replacement of interconnected systems, including organs Synthetic cells, tissue‐integrated devices, and progressive brain replacement
Ageing biomarkers and surrogate endpoints Screen and rejuvenate donor organs Monitor donor/recipient organ age and function Personalised replacement via organ‐specific ageing rates Mechanistic clocks dissecting cross‐tissue rejuvenation and age acceleration Novel molecular indicators, cell states, and cross‐organ interactions Longitudinal omics and functional monitoring
Transplantation therapies Optimise organ combinations and replacement cycles Resist graft age assimilation (study germ line, embryos and teratomas) Genome‐edited organs/cells to eliminate immunogenicity Personalised bioprinting that resists rapid ageing (e.g., receptor knockout) Replace fibrotic tissue and modulate inflammation Immune‐compatible, off‐the‐shelf stem cell biobanks
Bioengineering and novel replacement strategies Personalised therapies via biosensing and microchip (e.g., body‐on‐a‐chip) systems Bioreactor‐enabled large‐scale cell production Genes and regulatory elements from long‐lived and regenerative species Inducers maintaining/regenerating the body without needing replacement Organ recovery in injury models/highly regenerative species to uncover rejuvenation Advanced cryopreservation of cells, tissues, and organs Perfusion and rewarming technologies for preserving organ viability

*Note:* Selected barriers (a) and prioritised research avenues (b) to advance biological and synthetic replacement‐based ageing interventions and synergistic, multi‐targeted technologies for the removal, export, and replacement of molecular and organellar damage, aimed at durable, whole‐body rejuvenation.

**TABLE 2:** Predicted advances and long‐term vision for replacement‐based ageing interventions.

Projected advances for replacement‐based ageing interventions
Systemic and durable rejuvenation Synthetic technologies removing or exporting many types of molecular and organellar damage Replacement integrated with damage removal not reliant on endogenous cellular processes Preventive multi‐system replacement Inducers maintaining/regenerating tissues without requiring replacement Progressive brain replacement, synapse‐level mapping, and tissue‐integrated devices Scalable whole‐body replacement strategies Hybrid biological–synthetic replacement across organ systems
Ageing biomarkers and surrogate endpoints Early detection of damage and functional decline Personalised prioritisation of damage and systemic alterations Identification of organ “minimum units” whose replacement yields systemic benefits Cross‐intervention omics, functional readouts, and ageing clocks Long‐term monitoring (efficacy, durability of rejuvenation, and safety) Novel molecular, cellular, and physiological state indicators
Transplantation therapies Allogeneic/xenogeneic multi‐organ transplantation with low immunogenicity Rapid, personalised bioprinting of tissues and organs Preventive fibrotic tissue replacement and inflammation modulation Replenish molecules, organelles, and cells necessary for robust, systemic rejuvenation Durable resistance to graft age assimilation Enhanced plasma‐based interventions (e.g., exercise‐mimetic plasma)
Bioengineering and novel replacement strategies Gene replacements informed by long‐lived and highly regenerative species Advanced, immunocompatible cell therapies (including CAR‐T, CAR‐M, and synthetic cells) Preventive, off‐the‐shelf cell therapies maintaining tissues and enhancing regenerative capacity Bioreactor‐enabled production of patient‐specific cell populations Reliable vascularisation of large, engineered tissues, and solid organs Regeneration guided by regenerative organisms (e.g., hydra, axolotl, spiny mouse)

*Note:* By the end of the 21st century, replacement‐based ageing interventions could achieve systemic, durable rejuvenation of human tissues, organs, or whole bodies, and prevent or reverse age‐related diseases. These approaches could also enable integrated strategies that combine multi‐targeted, synthetic, and selective damage‐removal and export technologies, replacement, regeneration, and maintenance of biological systems.

## 2 Multi‐Tissue Replacement as a Preventive Ageing Intervention

Sierra Lore (Buck Institute and University of Copenhagen) opened the workshop by suggesting that replacement might be the single most effective intervention for extending healthy lifespan, questioning why there is insufficient focus on the development of preventive replacement strategies aimed at slowing ageing and rejuvenating healthy bodies (Lore et al. 2025). This research direction may have been hampered by a tendency to overestimate scientific development in the short term (e.g., 5 years), while underestimating the possibilities several decades from now (e.g., 75 years). Recalling the sparsity of replacement technologies 75 years ago can help us envision that in 2100, bioprinted organs, immune system resets, synapse‐level brain mapping, and reconstruction, and tissue‐integrated devices, could be the foundation for healthy longevity (Table 2, Figure 2a,b).

Vadim N. Gladyshev (Harvard Medical School) emphasised the importance of assessing long‐term effects on the biological age and function through tissue age assimilation. V.N.G. demonstrated a set of examples where age assimilation happens, and noted that despite encouraging results, many questions and critical barriers remain, including age incompatibility and host immune cell infiltration. To counteract the rapid age assimilation, it will be important to understand the barriers preventing age assimilation in the biological system. Organs can only be partially rejuvenated due to irreversible age‐related changes, such as extracellular matrix (ECM) remodelling. Considering the number, sizes, and functions of tissues could help ensure sustained rejuvenation (Tables 1 and 2).

Bohan Zhang (Harvard Medical School) outlined the potential of using biomarkers of ageing to quantitatively dissect rejuvenation mechanisms in models such as heterochronic parabiosis to reveal novel organ rejuvenation strategies (Tables 1b and 2). B.Z. and colleagues discovered that 3‐month exposure to young circulation extended lifespan in old mice, and the DNA methylation age remained reduced in liver and blood 2 months after detachment (Zhang, Lee, et al. 2023). The transcriptomic signature correlated with calorie restriction and growth hormone deficiency, but was negatively correlated with the ageing signature. When asked about how to obtain more mechanistic insights, B.Z. mentioned that another feasible strategy would be to study how organisms recover from adversity (Table 1b).

## 3 Clinical Applications of Bioengineering‐Based Replacement Therapies

Anthony Atala (Wake Forest Institute for Regenerative Medicine) described replacement as a most promising solution to tackle ageing. The Wake Forest Institute for Regenerative Medicine (WFIRM) is working on 40 tissues and organs to develop replacement‐based cell therapies, engineered tissue and organ replacements, and enabling technologies, with 17 applications currently in patients. For example, one clinical trial introduces cells into the urinary sphincter to treat urinary incontinence, while another trial treats shoulder rotator cuff injury using satellite cells, which decreased fat infiltration and increased muscle‐to‐fat ratios. In a further trial, chondrogenic priming of progenitor cells (Bolander et al. 2023) obtained from end‐stage (stage 4) knee osteoarthritis patients regenerated their articular cartilage and bone.

WFIRM recently began treating patients with multipotent stem cells isolated from amniotic fluid or the placenta (De Coppi et al. 2007). The cells are anti‐inflammatory, rapidly expand, lack the tumourigenic potential of embryonic or induced pluripotent stem cells, and incorporate better into tissues than adult stem cells. When asked about potential immunological challenges, A.A. replied that a cell bank of ~20,000 unique specimens would provide over 80% of the population with a genetic match (Table 1b).

WFIRM combines 60 types of biomaterials to repair structural defects and recreate tissues when cell therapies are insufficient (Table 1b). A.A. led the first human implantation of an engineered organ, a bladder, informing the subsequent development of others, such as engineered tubularised urethras and vaginal organs that remained functional long‐term. Bioreactors prevent tubular structure collapses and exercise engineered heart valve leaflets and blood vessels, which enabled a carotid artery transplantation. Solid organs, such as the liver, kidney, and heart, are the most complex and time‐consuming organs to engineer. Based on a project that started in 1990, engineered kidney therapies transplanted in an ongoing phase 3 clinical trial appear to provide enough function to prevent dialysis in patients.

A 14‐year‐long 3D bioprinting project enabled the bioprinting of skin with six primary cell types (Jorgensen et al. 2023), completion of NASA's Vascular Tissue Challenge, and extensive work on bioprinting in space. Six bioprinted tissues can be maintained within a single body‐on‐a‐chip system, facilitating drug development, physiological modelling, and personalised medicine. WFIRM has multiple clinical trials using tumour‐on‐a‐chip technologies for cancer treatment predictions. Such microchip technologies provide versatile platforms for developing and testing replacement‐based ageing interventions (Table 1b, Figure 2b).

## 4 Lineage Tracing and Cross‐Species Studies Inform Replacement Strategies

Aiming to create cell types for tissue replacement, Kyle M. Loh (Stanford University) is mapping the timing and combinations of extracellular signals to differentiate human pluripotent stem cells (hPSCs) into two dozen cell types. For instance, K.M.L.'s laboratory has developed methods to differentiate hPSCs into blood vessel cells (Ang et al. 2022) (with the long‐term goal of vascularising engineered human organs for organ transplantation) and haematopoietic progenitors (Fowler et al. 2024) (with the long‐term goal of blood and immune system replacement in vivo).

K.M.L. is also developing methods to differentiate hPSCs into forebrain, midbrain, and hindbrain neurons in vitro, thereby laying the foundation for future brain cell replacement (Tables 1 and 2). Their work challenged the notion that the entire brain emerges from a common progenitor, and instead suggested that separate progenitors generate the forebrain and midbrain versus the hindbrain in vivo (Dundes et al. 2025). The discovery of the hindbrain's in vivo progenitor inspired methods to differentiate hPSCs into hindbrain neurons in vitro, which are essential for breathing, sleep, movement, and other life‐sustaining functions in vivo (Dundes et al. 2025).

When asked whether self‐identity could be retained following hindbrain replacements, K.M.L. remarked that it might be possible, because the hindbrain controls autonomic functions such as wakefulness, sleep, eating, and breathing (Tables 1 and 2).

Vera Gorbunova (University of Rochester) offered insights into gene replacement as a complementary approach to organ replacement, particularly of the brain, to treat or prevent diseases and extend lifespan (Table 1b). V.G. identified several gene replacement candidates using comparative biology approaches to study animals with unique traits or long lifespans. Accumulation of high‐molecular‐mass hyaluronan in the naked mole rat underlies its exceptionally high cancer resistance (Tian et al. 2013), and overexpression of its hyaluronic acid synthase 2 gene in mice increased hyaluronan levels, reduced inflammation in many tissues, reduced cancer incidence, improved healthspan, and extended lifespan (Zhang, Tian, et al. 2023).

Overexpression of the bowhead whale CIRBP protein enhanced DNA repair efficiency and fidelity in human cells (Firsanov et al. 2025), and CIRBP extended lifespan and increased X‐ray resistance in *Drosophila melanogaster* , which lacks a homologue.

A comparative study across 18 rodent species showed that SIRT6 double‐strand break repair activity correlated with maximum species lifespan (Tian et al. 2019), and unpublished data indicates that transient overexpression of SIRT6 or SIRT6 delivered as gene therapy has anti‐ageing effects in human cells and in mice (Table 2).

## 5 Future Trajectories of Replacement in Longevity Science

In the panel discussion, moderated by S.L., V.N.G. reasoned that future studies should investigate why the lifespan extension of heterochronic parabiosis is limited and why the rejuvenation effects diminish over time (Tables 1 and 2). Gladyshev's laboratory is now exploring whether increasing the duration and cycle number of parabiosis amplifies the effects. Thomas Rando (University of California, Los Angeles) also observed diminishing effects and suggested that a continuous supply of young blood might be needed to sustain parabiosis‐induced epigenetic reprogramming. Tony Wyss‐Coray (Stanford University) has observed that plasma transfusion can reproduce many of the molecular effects of parabiosis, and that others have shown that young macrophages are beneficial in injury models. The T.W.‐C. lab demonstrated that cell types respond differently to therapeutic plasma exchange, and it is thus important to dissociate the most responsive cell types, which appear to be HSCs and hepatocytes (Table 1b).

Eric Verdin (Buck Institute) set up a therapeutic plasma exchange trial that reduced biological age in humans (Fuentealba et al. 2025), inspired by Irina Conboy's studies on plasma removal and albumin infusions. E.V. emphasised the need to do more work on identifying the most informative ageing clocks in such contexts, the optimal infusion material, and the frequency of plasma exchange, noting that too many plasma transfusions impeded biological age reduction (Fuentealba et al. 2025). T.R. remarked that plasma transfusion from exercised animals produces effects similar to those of young blood, and T.W.‐C. mentioned a Norwegian clinical trial in which AD patients are receiving exercised plasma from young, fit, healthy adults (Tari et al. 2022). V.N.G. noted that it will also be important to assess the long‐term effects of plasma exchange, given the diminishing effects observed upon parabiosis (Table 2).

S.L. noted that identifying individual differences in the minimum unit of replacement (e.g., a set of organs) required for strong rejuvenation would be informative, and that this could be tested using ageing biomarkers (Table 2). A.A. observed that fibrosis due to inflammation and cell death is a major culprit in organ failures and diseases. Thus, it will be key to replace fibrotic tissue, a major aim of cell replacement therapies, and to target inflammation, while considering the major role that stem cells appear to have. A.A. stated that an important challenge linked to preventing ageing is to implement early diagnosis and application of replacement interventions before approximately 90% of the organ has become dysfunctional, because organs such as kidneys, lungs, and blood vessels tend to fail at that point (Tables 1 and 2).

In a discussion about the early decline of the immune system, E.V. mentioned that there are many promising treatment avenues, but that the mortality of HSC replacement is currently high and not yet near the level of safety and minimal complications that would make it feasible as an ageing intervention.

S.L. asked the panellists to predict how replacement interventions might be used to target ageing in 2100. V.N.G. envisaged that the whole body (without the head) could be replaced, and referred to promising visions by Jean Hebert, who pioneers partial brain replacement, and biotechnology companies such as Retro Bio (Table 2, Figure 2b). A.A. emphasised that the main shift will be to consider and target the unified systems that lead to ageing and disease progression, rather than taking biopsies and regenerating separate organ systems upon injury or disease. Instead of fully relying on replacement, there may be inducers of cells that could be introduced into the body to promote regeneration, depending on an improved understanding of cell interactions, growth, and differentiation. Similarly, T.W.‐C. projected that the further identification of molecular indicators of cell and physiological states, including protein modifications, will enable strategies that maintain organs and cells, without the need for replacement (Table 2, Figure 2b).

T.R. predicted that there will be a phase of cell therapies and engineered vascularised tissues and organs before we can detect disease processes and intervene early enough to rely on maintenance strategies only. Akin to the gene replacement strategies that V.G. described, E.V. sees great potential in many interventions based on CAR‐T cells to enhance the immune system. T.R. suggested that off‐the‐shelf cell therapies, including CAR‐T cells, could be used to prevent diseases whose underlying mechanisms remain poorly understood, such as neurodegenerative diseases (Figure 2b). E.V. emphasised the importance of acknowledging the potential for many unforeseen advances that may redefine what is possible, quoting Niels Bohr: “It is difficult to make predictions, especially about the future.”

In the Q&A, B.Z. raised the prospect of permanently replacing human tissues with engineered tissues recapitulating the regenerative capacity of highly regenerative species such as hydra. V.G. emphasised that it is an interesting research direction and that several regenerative vertebrate animals can offer relevant insights, including the spiny mouse and the axolotl (Table 1b). T.R. noted that another solution could be to develop off‐the‐shelf therapies optimised for regeneration (Figure 2b). Addressing E.V.'s comment, K.M.L. stated that antibodies used to purge HSCs prior to transplantation are reducing the safety risks associated with donor‐recipient incompatibility. E.V. added that a better understanding of the immune system, alongside strategies such as genetic editing, will enable the mitigation of immunogenic complications associated with both allogeneic and xenogeneic transplantation (Table 2).

## 6 Key Takeaways

Recent discoveries have highlighted opportunities and challenges associated with biological and synthetic replacement‐based ageing interventions, which hold substantial potential to extend healthy lifespan beyond what can be achieved with conventional treatments that target relatively few age‐related changes (Table 2). There is an urgent need to develop preventive, immune‐compatible replacement strategies capable of inducing sustained, systemic rejuvenation in healthy individuals, rather than targeting isolated tissues or organs only after injury or disease has occurred (Figure 2a,b).

To maximise impact, it will be crucial to combine replacement therapies with the development of synthetic and bioengineering technologies that maintain and regenerate tissues, remove or export hundreds of forms of molecular and organellar damage simultaneously from cells, and target forms of irreversible damage that replacement alone cannot reverse, including extracellular matrix remodelling (Figure 2a,b). Such interventions are predicted to act synergistically with replacement strategies and could enable rejuvenation of tissues and organs both before and after transplantation. In particular, enhancing endogenous or synthetic cellular export pathways for damaged molecules and organelles could broadly reduce the accumulation of damage originating both within replaced cells and systemically from other tissues (Figure 2a).

Key translational hurdles for replacement‐based ageing interventions include tissue damage, host immune cell infiltration, and rapid ageing observed following transplantation of young organs, and the potential disruption of self‐identity during progressive brain replacement (Table 1a). Long timelines required to verify the benefits of replacement interventions in clinical trials, together with the lack of integrated frameworks that could achieve whole‐body or cross‐organ rejuvenation, are further complicated by the lack of validated molecular and functional biomarkers for accurate quantification of age and damage. Thus, progress in translating personalised replacement interventions depends on the identification of robust ageing biomarkers and surrogate endpoints capable of predicting long‐term functional outcomes across cell types, organs, and systems, including the extent and durability of rejuvenation (Table 2, Figure 2b). Characterising damage clearance by replacement interventions may inform the development of next‐generation inducers and synthetic interventions for maintaining and regenerating tissues and organs without the need for replacement (Figure 2a,b).

The recommended research directions and challenges identified in this Perspective establish a roadmap for prioritising research and innovation to translate these insights into systemic, whole‐body rejuvenation interventions aimed at substantially extending healthy lifespan.

## Author Contributions

**Bjorn Fraser Olaisen** wrote the manuscript. All authors reviewed, edited, and shared key points. **Vadim N. Gladyshev**, **Bohan Zhang**, **Anthony Atala**, **Kyle M. Loh**, and **Vera Gorbunova** presented their work. **Thomas A. Rando**, **Tony Wyss‐Coray**, **Eric Verdin**, **Anthony Atala**, and **Vadim N. Gladyshev** took part in the panel discussion moderated by **Sierra Lore**, who organised and facilitated the Replacement in Aging workshop. **Alexander Zhavoronkov**, **Morten Scheibye‐Knudsen**, and **Daniela Bakula** are the main organisers of the ARDD conference, which enabled the Replacement in Aging workshop.

## Funding

The authors have nothing to report.

## Conflicts of Interest

The authors declare no conflicts of interest.

## Data Availability

The authors have nothing to report.
